# Promoting rational antibiotic prescribing for non-complicated infections: understanding social influence in primary care networks in Germany

**DOI:** 10.1186/s12875-020-01119-8

**Published:** 2020-03-14

**Authors:** Regina Poss-Doering, Martina Kamradt, Katharina Glassen, Edith Andres, Petra Kaufmann-Kolle, Michel Wensing

**Affiliations:** 1 Dept. of General Practice and Health Services Research, University Hospital Heidelberg, Im Neuenheimer Feld 130.3, 69120 Heidelberg, Germany; 2 aQua Institut, Maschmuehlenweg 8-10, 37073 Goettingen, Germany

**Keywords:** Primary care networks, Quality improvement, Appropriate antibiotics use, Social influence processes, Peer exchange, Mixed-methods

## Abstract

**Background:**

Primary care networks in Germany are formalized regional collaborations of physicians and other healthcare providers. Common goals are optimized healthcare processes and services for patients, enhanced communication, agency for professional concerns and strengthened economic power. In the ARena study (Sustainable reduction of antibiotic-induced antimicrobial resistance), 14 primary care networks in two federal German states aimed to promote appropriate antibiotics use for acute non-complicated infections by fostering awareness and understanding. Factors related to the role of primary care networks were to be identified.

**Methods:**

For this study, audio-recorded telephone interviews were conducted with physicians, non-physician health professionals and stakeholder representatives. Pseudonymized verbatim transcripts were coded using thematic analysis. In-depth analysis was based on the inductive categories ‘social support’, ‘social learning’, ‘social normative pressures’ and ‘social contagion’ to reflect social influence processes. Data generated through a survey with physicians and non-physician health professionals were analyzed descriptively to foster understanding of the networks’ potential impact on antibiotic prescribing.

**Results:**

Social influence processes proved to be relevant regarding knowledge transfer, manifestation of best-practice care and self-reflection. Peer communication was seen as a great asset, the main reason for membership and affirmative for own perspectives. All interviewed physicians (*n* = 27) considered their network to be a strong support factor for daily routines, introduction of new routines, and continuity of care. They utilized network-offered training programs focusing on best practice guideline-oriented use of antibiotics and considered their networks supportive in dealing with patient expectations. A shared attitude combined with ARena intervention components facilitated reflective management of antibiotic prescribing. Non-physician health professionals (*n* = 11) also valued network peer exchange. They assumed their employers joined networks to offer improved and continuous care. Stakeholders (*n* = 7) expected networks and their members to be drivers for care optimization.

**Conclusion:**

Primary care networks play a crucial role in providing a platform for professional peer exchange, social support and reassurance. With regards to their impact on antibiotic prescribing for acute non-complicated infections, networks seem to facilitate and amplify quality improvement programs by providing a platform for refreshing awareness, knowledge and self-reflection among care providers. They are well suited to promote a rational use of antibiotics.

**Trial registration:**

ISRCTN, ISRCTN58150046. Registered 24 August 2017.

## Background

In Germany, primary care networks (PCNs) are comprehended as an important vehicle for optimizing local primary and cross-sectoral care for the benefit of patients, physicians and stakeholders [1]. Similar to communities of practice [2], these formalized collaborations of physicians and other healthcare providers interact regularly and share patients, set goals, standardize treatment and care, discuss concerns and attend continuing education. In addition, networks can provide economic advantages, such as power in negotiations with health insurers. Currently, more than 400 PCNs with up to 100 physicians aim to act as coordinators and moderators in regional primary care in Germany, bringing together general practitioners, ambulatory medical specialists, nursing homes, hospitals and self-help support organizations to optimize quality and efficiency of care [3, 4]. Differing in strategic alignment and ranging from small groups of loosely connected single physicians to professionally managed organizations with 20 to 100 practices, these networks offer the entire range of primary care [5]. All PCNs aim to provide above-average quality and outcomes of care as well as high patient satisfaction with care [3].

The cluster randomized trial ARena (Sustainable reduction of antibiotic-induced antimicrobial resistance, 2017–2019, trial registration: ISRCTN, ISRCTN58150046) intends to promote the rational and appropriate use of antibiotics for acute non-complicated infections in primary care in Germany [6, 7]. Using a multifaceted strategy with multiple interacting intervention components, ARena addresses physician, primary care team and patient knowledge and attitudes about the use of antibiotics [6]. ARena is embedded into 14 PCNs across the German federal states of Bavaria and North-Rhine Westphalia. Essentially, the promotion of a rational, reasonable use of antibiotics is based on fostering awareness for and understanding of the growing challenges of antimicrobial resistances (AMR) by effective communication, education and training addressed to PCNs physicians and care teams as well as the regional public. As ARena is not completed yet, assessment of intervention effectiveness is currently pending. With the aim of providing insights into determinants of practice regarding a rational use of antibiotics and to propose explanations referencing identified influences and mechanisms of action, ARena is accompanied by a process evaluation to explore factors and processes leading to impacts on antibiotic prescribing patterns [6].

Insights into the development of PCNs and their contributions to regional improvements in patient care already have been gained, yet predominantly are limited to specific single networks [3, 8–10]. Research found that PCNs can effectively shape healthcare by acting as a driver for innovation and optimized performance, especially where effective network strengths meet potentially new approaches to care [5]. Also, physician peer networks have emerged as a potentially important factor influencing medical practice [11]. Thus, being part of such a network might support the adoption of specific behaviors. This support may be attributed to social contagion as an influencing process in which network members are impacted by each other in their adoption decisions [12]. Social contagion theory suggests that human behaviors and traits can spread in social networks [13], assuming this is promoted by various behavioral mechanisms, such as imitation, role modelling and persuasion [12]. As interacting physicians in networks likely share their beliefs, ideas and experiences with each other, the interpersonal information exchange may influence practice patterns [14] and turn peer influence into a potential driver of physicians’ practice styles [15]. Studies of communities of practice and physician peer networks have identified differences in care patterns and patient outcomes based on peer connections [15–18] with regards to therapy uptake [16], or the adoption of technology [15], but have not focused yet in-depth on how physicians might have an impact on each other [11]. The role PCNs might play in supporting rational prescribing and use of antibiotics for non-complicated infections is yet unknown. The aim of this study was to explore factors and processes attributed to the network’s contribution to improving antibiotic prescribing in the ARena project.

## Methods

### Design

ARena was a three-armed cluster randomized trial, implemented in 14 PCNs in two German federal states (Bavaria and North Rhine-Westphalia). Intervention components included e-learning on communication, quality circles and data-based feedback for physicians and care team members, public information campaigns, performance-based additional reimbursement, a computerized decision support system and culture-sensitive information material for patients in print and digital format on tablet computers to be used in waiting areas. The moderated quality circles were held for all participating PCNs at four different times over the course of the intervention to facilitate discussion, review and critical assessment of clinical practice. Key issues related to care quality and the rational use of antibiotics regarding respiratory tract infections, urinary tract infections, pneumonia and multi-resistant pathogens were discussed in groups of healthcare professionals. Standard care is reflected by an added cohort based on claims-data [6].

In a mixed-methods approach, the process evaluation used open-ended, semi-structured interviews with primary care physicians (general practitioners, ear-nose throat specialists, urologists and pediatricians), non-physician health professionals – comparable to medical assistants (MA) in USA [19] - and stakeholder representatives (PCNs management, health insurance and patient representatives). Also used were a one-time socio-demographic survey of interviewees and study-specific questionnaires given to all physicians included in the intervention and all MAs in one of the three intervention arms at three different points in time. Different interview guides were developed for the three groups of interviewees based on a literature review and pre-defined research questions. The first interview in each group served as a pilot so minor adjustments could be made where considered appropriate. All qualitative data generated from interviews with physicians, MAs and stakeholders, field notes and the socio-demographic survey were included for analysis. In addition, data referencing PCNs were extracted from two sets of items in the first and second of three questionnaires (T0, T1) and included (Supplementary File 1 provides a translated version of the complete questionnaire.). Study-specific questionnaire construction was guided by the Theory of Planned behaviour [20]. A pre-test was conducted with 4 non-participant physicians to ensure relevance and clarity of all items. Thus, a broad thematic spectrum could be analyzed to assess and understand factors relevant to the role of PCNs regarding support for rational use of antibiotics for acute non-complicated infections.

### Study population for interviews

Qualitative interview data were collected beyond the point of saturation until deviant observations and consistency of findings allowed to assess data sufficiency. Applying a purposive strategy, a sample of 45 participants were recruited by the ARena study team at the Department of General Practice and Health Services Research, University Hospital Heidelberg between March and May 2018. Potential recruits were all physicians and MAs participating in ARena as well as managerial stakeholder representatives of participating PCNs and health insurance providers, association of statutory health insurance physicians and of a self-help organization.

All recruits had to be at least 18 years of age, legally fully competent and in fluent command of German. The recruitment procedure aimed at even distribution regarding gender and intervention groups. To be included in the process evaluation, all interested parties meeting the inclusion criteria received printed material as well as a phone call to provide further information, and were obliged to return a signed letter of intent for participation in the interview.

Out of 303 eligible physicians, 40 physicians in each of the three intervention groups (*n* = 120) were invited by e-mail via the aQua Institute, Goettingen, to participate in an interview. After 3 weeks, a reminder was e-mailed. Due to a disproportionately high number of recruited participants from Bavaria, an additional reminder was e-mailed to a random sample of 11 physicians located in North-Rhine Westphalia after 12 weeks. Out of 84 eligible medical assistants, 25 were contacted. Calculation of the number of contacted potential recruits was based on anticipated response rates and previous experiences. It also aimed to approximate the targeted number of interviews as defined by the study protocol [6]. All stakeholder representatives (*n* = 7) were known contacts of the aQua Institute and therefore personally addressed by aQua Institute staff via e-mail or letter. All invitees were sent a personalized cover letter, supplemented by information specific to study and process evaluation details and a feedback form to be returned by e-mail or fax to declare interest in participation.

### Study population for survey

All physicians participating in the intervention groups were invited to take part in the survey (T0 *n* = 303, T1 *n* = 312, T2 *n* = 292). Also, MAs employed at eligible participating primary care practices allocated to intervention arm B, were invited to take part in the survey (T0 *n* = 84, T1 *n* = 88, T2 *n* = 85). E-mail reminders were sent out after 4 weeks to increase the response rate. The study-specific questionnaires T0, T1 and T2 were to be sent out at three points in time over the course of the study. Response rates among physicians were 75.5% (T0), 64% (T1) and 63.3% (T2), and for MAs 95% (T0), 64.2% (T1) and 68.2% (T2).

## Data collection and analysis

### Interviews

Between April and June 2018, all physician interviews (*n* = 27) were conducted and digitally audio recorded by three researchers (RPD, MK, AS) of the study team at the Department of General Practice and Health Services Research, University Hospital Heidelberg. A semi-structured interview guide was followed to gain insights into typical practice referring to antibiotic prescribing and consideration of patient preferences, implications of the quality improvement program and its’ intervention components for patient care, general contextual factors and the role of the PCNs in particular. Two researchers (RPD, MK) conducted and audio recorded all interviews with medical assistants (*n* = 11) in April and May 2018. Besides the aforementioned topics, the interview guide specifically focused on MA perspectives and experiences. All stakeholder interviews (*n* = 7) were conducted and audio recorded by the same two researchers in April and May 2018. Here the interview guide was tailored to cover stakeholder expectations for the potential influence of the intervention components and perspectives on context factors as well as recommendations for the future use of antibiotics.

All interviews (*n* = 45) were conducted over telephone. Additional notes were taken to document participant suggestions with regards to aspects of intervention delivery. After data collection was completed, pseudonymized verbatim transcripts were coded applying a thematic framework analysis [21] based on the Tailored Implementation for Chronic Disease (TICD) framework which uses 7 domains to classify determinants of implementation (Guideline factors, Individual health professional factors, patient factors, Professional interactions, Incentives and Resources, Capacity for organizational change and Social, political and legal factors) [22]. In compliance with the ARena study protocol [6], the pre-defined categories of the TICD were used to identify determinants of practice regarding potential changes in health professional practice concerning the appropriate use of antibiotics in acute non-complicated infections in primary care [6]. The interprofessional team of researchers (Public Health and Health Services Research) identified themes of interest deductively a priori from the TICD framework as well as inductively de novo from the data itself during the analysis. Two researchers (RPD, MK) coded all transcripts independently and iteratively using MAXQDA Analytics PRO 18 (Release 18.0.3). Divergent codings were discussed continuously to ensure intercoder congruity and to achieve the widest consensus possible. To enable a broader view, participant socio-demographic characteristics were analyzed descriptively using IBM SPSS Statistics Version 24. In an effort to facilitate understanding of the role and the mechanisms of influence of PCNs in promoting a rational use of antibiotics for acute non-complicated infections, an integration of the theoretical perspective on social influence processes was applied to further deepen data analysis and interpretation and to propose explanations referring to identified relevant factors.

### Surveys

Study-specific questionnaires were mailed to participants in January 2018 (T0), October 2018 (T1) and July 2019 (T2). All questionnaires focused on the implemented intervention components, relevant context factors, prescribing decisions and general perceptions regarding antibiotics. Additionally, T1 and T2 asked for interim and concluding assessments of the intervention components, respectively. Completed questionnaires were returned to and registered by the study team at the Department of General Practice and Health Services Research, University Hospital Heidelberg, between February and April 2018 (T0), November 2018 to January 2019 (T1), and July to September 2019 (T2). All completed questionnaires were digitalized and subsequently, data were transferred into IBM SPSS Statistics 24 for descriptive analysis. Data visualization was performed using Microsoft Excel 2010. Findings from the survey data are reported here with a focus on the potential contribution networks might provide regarding communicative thematic peer exchange and educative efforts geared towards care teams.

Table 1 outlines the data collection sources relevant for findings presented here.

**Table 1:** Data collection sources

Source	Physicians	Medical assistants	Stakeholders	Description
Interviews (n)	27	11	7	Over telephone
Socio-demographic questionnaire (n)	27	11	7	Paper based
Notes (n)	14	3	2	Paper based and electronically
Survey T0 (n)	229	80	–	Paper based
Survey T1 (n)	200	73	–	Paper based

## Results

### Overview

The results outlined below reflect the identified factors and processes of influence of PCNs in promoting a rational use of antibiotics for acute non-complicated self-limiting infections. A socio-demographic questionnaire was analyzed descriptively with regard to participant- and practice-level characteristics. A total of 27 physicians participated in the interviews (9 female, 18 male). Physician interview durations varied between 7:54 min and 62:50 min, with a mean of 28:14 min. MA interview durations ranged between 17:30 and 42:32 min (mean 26:53 min). All participating MAs were female (*n* = 11). Mean duration of stakeholder interviews was 30:15 min (range 16:28 to 44:42 min). Stakeholders had between 1 and 10 years of experience in their current position. On average, physician respondents in the survey were 55 years old, had 25 years of working experience and had been members of their PCNs for 10 years (Table 2).

**Table 2:** Characteristics of the interview sample and survey respondents (T0)

Interview participants	N	Phys	MA	Stakeholder	Total
Sex (f/m) n (%)	45	9/18 (33/66)	11 (100/0)	3/4 (43/57)	23/22 (59/41)
Age years (range) (mean)	45	43–66 (55.2)	20–60 (38.5)	31–63 (46.3)	31.3–63 (46.6)
Experience in current position years (range) (mean)	45	10–38 (26.07)	2–40 (19.3)	1–10 (5.85)	1–40 (17.09)
Working in general practice (%)	38	66.6	81.8	–	74.2
Part-time employment n (%)	4	1 (2.7)	3 (27.3)	–	4 (8.88)
Practice network member years (range) (mean)	27	2–23 (10.18)	–	–	10.18
Additional qualifications n	7	–	7	–	7
**Survey respondents (T0)**					
Sex (f/m) n (%)	304	229 (148/76)	80 (100/0)	–	228/76 (74/26)
Age years (range) (mean)	299	35–73 (54.4)	19–61 (38.7)	–	19–73 (46.5)
Working experience years (range) (mean)	306	5–48 (25.4)	1–40 (19.2)	–	1–48 (22.3)
Working in general practice (%)	309	75.3	76	–	75.6
Resident years (range) (mean)	220	1–41 (17.7)	–	–	220 (17.7)
Network member years (range) (mean)	207	0–28 (10)	–	–	10
Participating in network events times/year (range)	217	7.3 (0–50)	–	–	7.3 (0–50)

**Fig. 1:**
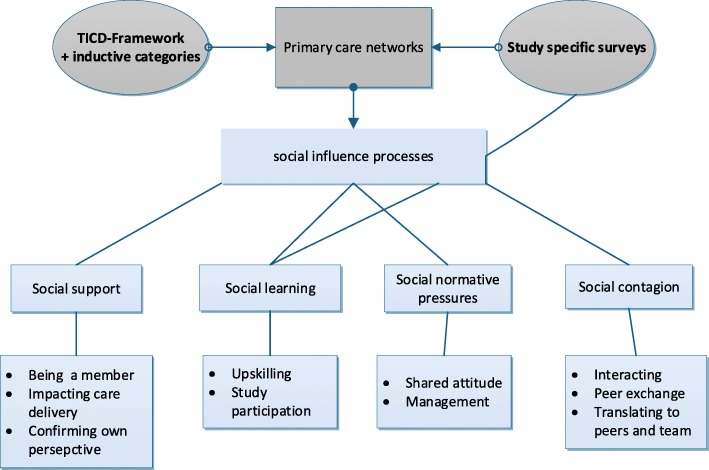
The analytical process applied in this study. Figure 1 describes the applied analytical process and shows the relevant sub-categories. Grey shapes represent deductive and inductive categories derived from the TICD and from survey items. Blue shapes represent identified social influence processes and related sub-categories

Findings from the qualitative data are reported regarding aspects explored within the inductive domain ‘Primary care networks’ and focus on social influence processes [23–25] identified during the analysis: perceived social support, social learning, social-normative pressures and social contagion. For illustration, extracted quotations have been included. All provided quotes were translated with due diligence and are referenced with participant number and transcript position. Additional quotes are provided as supplementary material (Supplementary Table 1). Figure 1 describes the analytical process and points out relevant sub-categories defined during the analysis.

### Social support

#### Being a network member

Generally, physicians and MAs felt their networks to be a strong support factor both for existing daily and implementing new routines. Data generated through surveys T0 and T1 showed that 74 and 59% respectively of physicians experienced support for new routines, 43 and 36% acknowledged network impact on prescribing decisions, guideline-oriented care (70.5 and 60%), and management of patient expectations (61.2 and 51%) and felt supported in pursuing shared-decision making (61 and 59%) (Table 3). Table 3 shows results from physician survey T0 and T1 on network participation.

**Table 3:** Physician perspective on network participation (T0, T1)

Participating in the network	Agree T0/T1 (%)	NeutralT0/T1 (%)	Disagree T0/T1 (%)
motivates guideline-oriented patient care	70.5/60	18.5/19	11/22
supports shared-decision making	60.8/59	19.8/24	19.4/18
supports managing patient expectations	61.2/51	21.6/30	17.2/19
supports implementing new routines	74/59	16.3/18	9.7/24
has an impact on antibiotic prescribing decisions	43.3/36	22.1/22	34.5/43

When asked about the significance of being a member in a primary care network in the interviews, participants shared clear ideas. Predominantly, cited aspects referred to being part of a group rather than working just as a single practice. Access to collective knowledge through active in person peer exchange was considered to be of high importance with regards to support in situations of uncertainty, complex cases and the synchronization of therapy decisions across disciplines. Physicians stated different reasons for initially joining their network including options for future prospects, further personal development, communicative peer exchange, upskilling and utilization of joint administrative structures.

Personally, I think that … it is the future, … the single practice, all alone without peer exchange, at least for me, it is definitely not a model I want to work in. Phys08, Pos. 56.

We have found a forum where we look at the big picture and that means, I don’t only look at pediatric topics but also at orthopedics, gynecology, dermatology and all other areas, and they, too, learn what is important for us, for life, for our perspective. Phys02, Pos. 20.

MAs felt that the network membership motivated them to support the physician (72% in T0 and 69,9% in T1), fostered dealing with patient expectations (68 and 71.3%), supported the implementation of new routines (71 and 68.4%) and had an impact on their part in patient care (59 and 60.3%). During the interviews, they shared physician views regarding future prospects, peer exchange and joint administrative structures. They also added their own perspectives referring to financial aspects and benefits for patient care. Stakeholder substantiated physician and MA perspectives, emphasized the importance of collaboration, communicative peer exchange and collective efforts of upskilling in networks. They also reflected on support of basic human needs.

Patient care, to begin with, exactly, so that patients are well supported, and I think, I will be honest, it might also be a question of billing. MA06, Pos. 44.

… makes you somewhat happier, fulfills your own expectations and of course motivates you, when you can enter into dialog and, as a bonus, can present yourself and what you can do really, really well, to the network, and you can collect everybody’s experiences – when this exchange happens. Sh04, Pos. 84.

### Impacting care delivery

Regarding care delivery, participants saw network membership as an asset and a decisive factor for quality management, both in the study context and beyond. Continuity of care within the network was seen as supported by more and closer peer contacts, simplified peer exchange, usage of shared electronic patient records, fast appointment scheduling and patient transfers to medical specialists.

Improved care services for the patients, not only in the ARena study, but in general … MA03, Pos. 75.

… important to me that I communicate with others and I believe, working in the network contributes to quality management in the long run … Phys23, Pos. 54.

Mutual communication is top priority, in my opinion, this is underestimated, and through such networks where you are forced to communicate with each other, this is promoted immensely, and all involved benefit and of course not least the patients. Sh04, Pos. 70.

### Confirming own perspective

Social support through communicative peer exchange was also seen as affirmative to own perspectives regarding importance of specific topics or course of own action.

This peer exchange is extremely important and contributes a lot to self-confidence, I presume. Phys23, Pos. 56.

Exactly these discussions happen in these networks, … What does this mean? How do I accompany patients, take care of them, how can I conduct conversations? To support each other in finding a way and entering into discussion … Sh02, Pos. 22.

### Social learning

#### Upskilling

Participants reported structured approaches of regularly offering upskilling events by their networks and taking part in a range of medical and organizational training programs such as quality and efficiency circles for MAs and physicians, workshops, case studies, and journal clubs. These learning opportunities were considered to be supportive in terms of providing new knowledge and peer exchange opportunities in a protective setting at the same time. Participants also felt supported by provided information components on evidence-based practice and provision of patient information.

We regularly meet in quality circles, I think that is quite good, and do case reviews … I find this very pleasant in the network, because you know each other and dare to come out of your shell … Phys14, Pos. 44.

### Participating in ARena

Physicians reported taking part in ARena-specific quality circles focusing on best practice guideline-oriented use of antibiotics and finding opportunity there for thematically relevant peer exchange within the network. Survey data (T0 and T1) showed that 83.4 and 86% of the participating physicians used the offered trainings on guideline-oriented antibiotics therapy and 71.5 and 72% acknowledged that peer exchange about antibiotic prescribing for acute non-complicated infections was provided within their network (Table 4). There was indication of a heightened alertness to quality improving mindful antibiotic prescribing as a result of the PCNs’ study participation.

**Table 4:** Results from surveys T0 and T1 referring to training and peer exchange

In my primary care network	Agree T0/T1 (%)	Neutral T0/ T1 (%)	Disagree T0/T1 (%)
… antibiotics therapy is discussed	89.5/86	8.8/10	1.7/4
… peer exchange about guideline-oriented antibiotics therapy is offered	79.9/79	14.5/11	5.6/10
… exchange about antibiotic prescribing routines for non-complicated infections is possible	71.5/73	18.4/16	10.1/11
… there are conventions about antibiotics for non-complicated infections	65.8/72	21.5/16	12.7/12
… training on guideline-oriented antibiotics therapy is offered	89/75	6.6/18	4.4/7
… I participated in training on guideline-oriented antibiotics therapy	89/87	6.6/9	4.4/4

### Social-normative pressures

#### Shared network attitude

Participants made references that pointed to a shared network attitude which included a common interest in evidence-based guideline-oriented care, mutual support in situations of uncertainty or locum care, long existing memberships, and a general sense of community. Financial aspects and ties to pharmaceutical manufacturers were negated. However, pointing to potential selection processes, it was also mentioned that PCN members needed to be willing to implement changes. This was reflected in survey T0 data, where 90% of the physicians indicated they had implemented changes in their practices during the past two years. Aspects of social integration and in one case a perceived lack of it were contemplated as well as potentially different circumstances in case of not being a network member.

So, I think, in this context [in the network], you generally mind your prescribing behavior and of course now in particular you mind your antibiotic prescribing, too. Phys22, Pos. 17 and 50.

Quality circles are obligatory. If one says ‘I can stay as I am’, he is not an adequate network physician, if you like, but this ultimately means change. Sh05, Pos. 56.

Thinking I would not be in the network and not have the [peer] exchange options, perhaps one would not have participated in the study at all, but overall, we probably would act differently and not attach so much importance to that. Phys22, Pos. 50.

### Management

Generally, the management teams of PCNs were seen positive and active in aiming for a strong common appearance, provision of information and training on new developments, evidence-based guideline-oriented care, and opportunities for peer exchange. In some cases, political involvement in contractual negotiations with health insurers were reflected on. One physician explicitly described the network manager as a go-ahead character who promoted digitalization efforts. This was the only mention of someone being considered an opinion leader regarding specific topics. One stakeholder saw network management in this role. Participants who were involved in network or stakeholder management gave their managerial viewpoint.

I am a network board member … it is important to me to absolutely provide know-how. Phys24, Pos. 38.

I don’t think physicians join a network and say ‘I want to practice a better medicine’, but this eventually has to be defined by the management, ... it’s an ambitious pledge and I think it only works in certain areas, … and this is where I see ARena. Sh05, Pos. 48.

… the network has a very honorable manager, a big IT promoter who … co-developed a very good data security concept that practices can implement quite safely. Phys09, Pos. 30.

### Social contagion

#### Interacting and peer exchange

Participants generally cited positive attributions regarding aspects of interaction and peer exchange and a potential impact that physicians might have on each other within the network. Some physicians expressed that they explicitly wanted to work in a PCN member practice to be able to make use of regular peer exchange. With regards to the role PCNs might play in supporting rational prescribing and use of antibiotics for non-complicated infections, they pointed to coordination of care-related aspects and the importance of regular peer exchange and interaction.

... I think we are more focused and faster, because I have the same thoughts as the cardiologist or the nephrologist … Phys27, Pos. 78.

Well, we have seen – as a single physician, there is no point in reading something. When we read as a group and the others say ‘yes, I do this, too’, ‘I will do it this way from now on, too, makes sense’, then I am more likely to implement it in my practice, because I know: OK, my peers do this, too. It’s an important effect. Phys17, Pos. 28.

That is why I explicitly looked for a practice where this [peer exchange] is possible. Phys08, Pos.56.

Potential influences of interacting in PCNs were also reflected critically with regards to antibiotic prescribing decisions, own attitude and using peer exchange windows of opportunity to exert educative influence. Physicians noted that sometimes networks lost members and it could be difficult to gain new ones. One physician described recruiting all physicians in specialist training in his practice. There was no indication of defined non-admission criteria. While physicians focused more on providing contagion in PCNs, stakeholder and MAs contemplated contagion processes.

It definitely has an effect for the patients, because we do evidence-based, scientifically sound medicine. But this has not changed because I am a network member, but we founded the network so we could infect others [with ideas]. Phys17, Pos. 36.

The network in the end does not have any influence on a physician’s decision, I think this remains with the physician. Sh01, Pos. 46.

We actually always have a team meeting when there is something new everyone should know about, and has been discussed [in a quality circle], and then we discuss it in the meeting and see how we can implement or improve certain things in our routines. MA01, Pos. 66.

#### Translating to peers and team

Physicians and MAs reflected on exchanging information and translating knowledge within their practices and networks in general as well as in the study context. While physicians again focused on providing information to peers, MAs took a more reciprocal perspective and took pride in translating knowledge back to their place of work.

On my end, I try to incorporate this ENT specialist view, to do genuine networking, to inform my colleagues, to give food for thoughts, reflect experiences I gain in my practice. Phys24, Pos. 38.

Sometimes it is very cool, there is this MA circle I joined, and you talk to peers, [about their routines], and then you are back at work and can narrate. MA11, Pos. 52.

Existing network structures regarding information dissemination and peer exchange were seen as promoting factors for knowledge translation and following up with new ideas. Participants reported using in-person meetings and direct phone calls, printed information media, video conferences and communication applications within their PCNs. There was no mention of fixed or loose subgroups in networks other than in connection to medical specialty or organizational level and related topics. There was little mention of communicative exchange between networks. Regarding ARena, participants emphasized their networks’ support for the implementation of the intervention components, following through with study participation and the potential to reach a larger group.

… I believe that network structures certainly … promote trying out even more, because both personal and administrative structures are in place. Sh07, Pos. 84.

Ok, one can easily say that without it [the network], we probably would not have followed through as consistently. Phys19, Pos. 62.

## Discussion

The ARena study is not completed yet and assessment of intervention effectiveness is currently pending. However, to assess the contribution of PCNs regarding promotion of a rational antibiotic prescribing for non-complicated infections, this study explored factors attributed to the network’s role with a focus on the impact physicians might have on each other. We found evidence for the role of various processes of social influence in this context: social support, learning, normative pressures and contagion. In particular, regular thematic peer exchanges as possible in quality circles and during continued training opportunities offered by PCNs, were emphasized by the participants as being of high value and support to them and considered to be one of the great assets the membership in a PCN entails. The contagion impact physicians might have on each other became apparent through acknowledgement of a higher likelihood of implementing new ideas and behaviors into practice after ascertaining that peers do so, too. Possibly, this implies that potential change resistances could be reduced by this contagion impact. These findings suggest that an interaction of professional peer exchange and a shared attitude within PCNs combined with intervention components as offered in the ARena study can promote a more reflected management of antibiotic prescribing. To achieve this, PCNs engaged in efforts of knowledge transfer and manifestation of best practice care by providing a platform for refreshing awareness and knowledge and facilitating self-reflection of prescribing practice for their members.

PCNs have been set up with specific purposes and for specific activities, such as quality improvement efforts. They aim for above-average quality of care and increased patient satisfaction. It may be that only individuals who share these aims are attracted to work in PCNs while others prefer unrestricted autonomy and therefore stay outside. This may imply selection processes that influence social influences and their impact in PCNs. If these fail to be considered adequately, social influence processes and potentially present opinion leadership are likely to be overestimated [26]. It is known that physicians influence each other, but the magnitude of peer influence is still poorly understood [27]. In this study however, participants attached general importance to social learning and the social support offered by their PCNs: Peer exchange was strongly emphasized as a major asset while there was hardly any mention of individual local opinion leaders. This is particularly notable as opinion leaders are a frequently examined aspect of social networks and considered to be a strong influence on other network members as they seem to amplify dissemination of ideas and information [28]. A Cochrane review confirmed that opinion leaders can promote evidence-based practice and reported a 10.5% increase in compliance when used as a strategy [29]. However, several studies failed to identify and clearly describe the role of clinical opinion leaders, particularly in ambulatory care settings [29, 30]. Physicians participating in this study only mentioned opinion leaders in their PCNs when asked general questions about PCNs management and scarcely regarding a rational use of antibiotics. Therefore, it is possible that opinion leaders in this specific setting and context would be comparably less effective, because hierarchical structures in general appear less suitable here [31]. Based on their training and socialization and high degree of autonomy in their professional practice, hierarchical leadership relations in PCNs may not even appeal to physicians or might cause conflicts in their original practice [32].

Taking a social network perspective enables understanding the diffusion of new ideas and behaviors and the process by which they are communicated over time [33]. It also facilitates understanding and evaluation of implementation processes [34] and potentially achieved behavior changes in network members. The adoption of new ideas and behaviors by individuals is strongly influenced by the quality and structure of their social network [34, 35]. Physicians tend to join more informal horizontal networks which are more effective for peer influence and constructing and reframing of meaning [36]. In such peer networks, interacting physicians likely share their beliefs, ideas and experiences with each other and these interpersonal exchanges may influence practice patterns [14]. Such social influence processes tend to homogenize ideas and behaviors within networks. Therefore, from a quality improvement and evidence-based practice perspective, guidance in the form of accepted educative programs, practice guidelines, or opinion leadership seems important to propel ideas and behaviors in the desired direction. In this study, physicians valued communicative peer interaction within their PCNs very highly and attributed less importance to opinion leadership. Participation in the study, evidence base and existing guidelines might have acted as strong motivation and push factors. Nevertheless, even if acting as opinion followers, the participants might still be viewed as opinion leaders themselves as they discussed a new behavior in peer exchanges, adopted it and carried it into their own practice.

Social influence processes, and particularly social contagion after communicative interaction, address the tendency of adopting ideas, behaviors and attitudes of connected individuals in networks [37, 38]. Contagion theory does not require an intent or even awareness to influence, only that communication takes place [39]. This study suggests that PCNs partly function as vehicles and amplifiers of social influence processes, such as imitation, role modelling and persuasion by providing a highly valued platform for communicative peer exchange. Thus, they can support efforts to promote a more reflected use of antibiotics.

Positive effects on quality of care and patient outcomes are considered to be present in PCNs when adequate resources and credible, efficient management are coupled with effective communication strategies and collaborative trusting relationships [40]. Close, trusting collaboration and communication among network members and increasing utilization of digital networking, paired with efforts of structured quality management and upskilling also constitute the base for the PCNs aim for above-average quality of care and increased patient satisfaction [3]. PCNs participating in the ARena study provided those resources and collaborative trusting environment required to support the reflected use of antibiotics regarding acute non-complicated infections. They hereby demonstrated that they potentially can play an even more important role in German healthcare. Nevertheless, to soundly identify influence processes at work, a follow-up regarding sustained behavior changes appears equally necessary as research into transposability of the identified factors to other types of primary care organizations.

### Strengths and limitations

For the ARena study and the implemented intervention components, the PCNs constituted an appropriate choice to deliver the implementation program as generally interventions delivered by a peer community can be expected to be more successful than those delivered by agencies less connected to program recipients [34]. In addition to the qualitative analysis, data referencing PCNs were extracted from surveys T0 and T1 and included to ensure a broad thematic spectrum could be analyzed within the process evaluation to assess and understand factors relevant to the role of primary care networks regarding support for rational use of antibiotics for non-complicated infections. The purposive sampling strategy supported the identification of individuals who were especially knowledgeable about and experienced in the phenomenon of interest and was chosen as it supports a detailed exploration and broad understanding of central themes, experiences, roles and behaviors [41]. Qualitative data was collected beyond saturation aspects until data sufficiency for this study was assessed. Reporting of this study follows the COREQ guideline and the CONSORT Statement where applicable [42, 43].

Some limitations of this study have to be reported. Participating PCNs were already susceptible to the topic and aware of implications which allows for a selection bias and possibly social desirability of shared perceptions. The influence of social processes might be overestimated as social selection processes were not considered in this study. All reported findings require cautious interpretation with regard to a complete and profound understanding of social influence processes. Combination and triangulation of all generated data is expected to provide further insights after concluding analysis.

## Conclusion

PCNs provide a platform for social influence processes regarding professional peer exchange, social support and reassurance. Their impact on antibiotic prescribing for acute non-complicated infections is based on facilitating and amplifying the delivered quality improvement program by refreshing awareness, knowledge and self-reflection among larger groups of care providers. Thus, they are well suited to promote a rational use of antibiotics.

## Supplementary information

**Additional file 1: Supplementary Table 1:** Additional quotes (translated from German).

**Additional file 2: Supplementary File 1**: Study-specific questionnaire (translated from German).

## Data Availability

All data generated and analyzed during this study are stored on a secure server at the University Hospital Heidelberg, Germany, Department of General Practice and Health Services Research. De-identified sets of the data collected and analyzed during this study can be made available by the corresponding author on reasonable request.

## Abbreviations

ARena: Sustainable reduction of antibiotic-induced antimicrobial resistance (German: Antibiotika-Resistenzentwicklung nachhaltig abwenden)
COREQ: Consolidated criteria for reporting qualitative research
G-BA: Federal joint committee (German: Gemeinsamer Bundesausschuss)
MA: Medical assistant
PCNs: Primary care networks
Phys: Physician
Sh: Stakeholder
