# Supplementary material for: Promoting rational antibiotic prescribing for non-complicated infections: understanding social influence in primary care networks in Germany
Source: BMC Fam Pract. 2020 Mar 14;21:51. doi: 10.1186/s12875-020-01119-8 (PMC7073012; doi:10.1186/s12875-020-01119-8)
Supplement: Supplementary file 1 — Additional file 1: Supplementary Table 1: Additional quotes (translated from German). [file 12875_2020_1119_MOESM1_ESM.docx]

**Supplementary Table 1: Additional quotes**

| Category | Sub-category | Quote |
| --- | --- | --- |
| Social support | Being a network member | “A team-feeling.” Phys01, Pos. 56  “To be together, we are stronger together!” Phys05, Pos. 28  “You are in a group, I think this is a little bit like our inner core that always looks for a partnership anywhere.” Sh04, Pos. 78  “Well, everybody has their own practice and will decide for themselves, but at least you have a platform for good exchange.” Phys27, Pos. 82 |
|  | Impacting care delivery | “Well, to ensure rural medical services and make sure patients can always come and we are here for them.” MA05, Pos. 56 |
|  | Confirming own perspective | “Such discussions are important to affirm the topic and confirm the own course of action. So, in a reflective discussion you learn how others act, how many use the same approach, do many use the same approach, where do you stand and fall in line, and this can be confirming or critical.” Phys21, Pos. 42 |
| Social learning | Upskilling | “We have solid structures in our network concerning continuing training.” Phys03, Pos. 54 |
| Social-normative pressures | Shared network attitude | “We are simply a care network that is not after the money.” |
|  |  | “We have been holding quality circles for the last 20 years anyway. We built a network on this. We are always the same group sitting together and we all work pharma-independently.” Phys17, Pos. 16 |
|  | Management | “… aiming to produce a certain corporate identity.” Phy04, Pos. 36 |
| Social contagion | Interacting and peer exchange | “I still think that the really decisive point is that we meet once a month. One evening, about three hours, and we can say – we are really proud to say - that 45 to 55 out of 65 physicians participate, so we can really achieve a lot, so we can really make sure almost all physicians are on the same page.” Phys04, Pos. 40  “Regarding ARena, the network event we had was particularly interesting to me and the discussion with the colleagues there.” Phys21, Pos. 38  “And sometimes, I consciously use this network to hint something to certain colleagues who show a modus operandi I consider inappropriate, without having to face instant harassment.” Phys02, Pos. 20 |
|  | Translating to peers and team | “… in this case here, of course, it works because of structures, e.g. a primary care network where you have participants who represent an intersecting subset of the overall network and when you communicate and inform via projects, of course knowledge and information can get translated to a larger part of the network.”  Sh03, Pos. 80  “My colleague brings the information [to us]. And in turn she takes our ideas, developments, questions back to the quality circle.” MA10, Pos. 318 |
