# Supplementary material for: Promoting rational antibiotic prescribing for non-complicated infections: understanding social influence in primary care networks in Germany
Source: BMC Fam Pract. 2020 Mar 14;21:51. doi: 10.1186/s12875-020-01119-8 (PMC7073012; doi:10.1186/s12875-020-01119-8)
Supplement: Supplementary file 2 — Additional file 2: Supplementary File 1: Study-specific questionnaire (translated from German). [file 12875_2020_1119_MOESM2_ESM.docx]

| **Quality improvement program in ARena** | | | | | |  |
| --- | --- | --- | --- | --- | --- | --- |
| **The e-learning module regarding patient-centered communication …** | **Disagree strongly** | **Disagree** | **Neutral** | **Agree** | **Agree strongly** | |
| ... was used by me |  |  |  |  |  | |
| … helps changing my communication with patients |  |  |  |  |  | |
| …helps with promoting the inclusion of patients in the decision process regarding the therapy |  |  |  |  |  | |
| ... motivates guideline-oriented patient care |  |  |  |  |  | |
| … makes the decision for or against antibiotic therapy easier |  |  |  |  |  | |
| … gives me confidence to handle patient expectations |  |  |  |  |  | |
| … impact my communication with patients |  |  |  |  |  | |
| **The quality circle meetings …** |  |  |  |  |  | |
| … were visited by me |  |  |  |  |  | |
| … help with taking therapy decisions for patients with non-complicated infections |  |  |  |  |  | |
| … motivate guideline-oriented patient care |  |  |  |  |  | |
| … make the decision for or against antibiotic therapy easier |  |  |  |  |  | |
| … give me confidence to handle patient expectations |  |  |  |  |  | |
| … impact my therapy decision for patients with non-complicated infections |  |  |  |  |  | |
| **Background information referring to a rational use of antibiotics …** |  |  |  |  |  | |
| … were read by me |  |  |  |  |  | |
| … impact my therapy decisions for patients with non-complicated infections |  |  |  |  |  | |
|  |  |  |  |  |  | |

| **The information material for patients ...** | **Disagree strongly** | **Disagree** | **Neutral** | **Agree** | **Agree strongly** |
| --- | --- | --- | --- | --- | --- |
| … are displayed in my practice |  |  |  |  |  |
| ... are read by my patients |  |  |  |  |  |
| … are helpful when discussing possible therapy options with patients |  |  |  |  |  |
| … motivate me to treat patients increasingly guideline-based |  |  |  |  |  |
| … makes the decision for or against antibiotic therapy easier |  |  |  |  |  |
| … give me a sense of security when handling patient expectations |  |  |  |  |  |
| … have an influence on my communication with patients |  |  |  |  |  |

| **The offered pay-for-performance in the study …** |  |  |  |  |  | |
| --- | --- | --- | --- | --- | --- | --- |
| … can promote a guideline-oriented care for patients with non-complicated infections |  |  |  |  |  | |
| … motivates guideline-oriented patient care |  |  |  |  |  | |
| … makes my decision for or against antibiotic therapy easier |  |  |  |  |  | |
| … gives me confidence to handle patient expectations |  |  |  |  |  | |
| … impact my therapy decision for patients with non-complicated infections |  |  |  |  |  | |
| **B. Contextual factors** | | | | | |  |

| **Structural conditions** (team, rooms) |  |  |  |  |  |
| --- | --- | --- | --- | --- | --- |
| … motivate me to treat patients increasingly guideline-based |  |  |  |  |  |
| … support me in taking shared therapy decisions with the patients |  |  |  |  |  |
| … support me in handling patient expectations regarding the prescription of antibiotics |  |  |  |  |  |
| … are helpful when implementing new routines in the practice |  |  |  |  |  |
| … impact the amount of time available to me per patient |  |  |  |  |  |
| … impact my decision to prescribe antibiotics |  |  |  |  |  |

| **Existing processes and organizing processes in the practice …** | **Strongly disagree** | **Disagree** | **Neutral** | **Agree** | **Strongly agree** |
| --- | --- | --- | --- | --- | --- |
| … motivate guideline-oriented patient care |  |  |  |  |  |
| … support me in taking a shared the therapy decision with the patients |  |  |  |  |  |
| … supports me in managing patient expectations regarding the prescription of antibiotics |  |  |  |  |  |
| … are helpful with implementing new routines in the practice |  |  |  |  |  |
| … impact the amount of time available to me per patient |  |  |  |  |  |
| … impact my decision to prescribe antibiotics |  |  |  |  |  |

| **Participating in the network …** |  |  |  |  |  |
| --- | --- | --- | --- | --- | --- |
| … motivates guideline-oriented patient care |  |  |  |  |  |
| … supports shared-decision making |  |  |  |  |  |
| … supports managing patient expectations regarding the prescription on antibiotics |  |  |  |  |  |
| … supports implementing new routines |  |  |  |  |  |
| … has an impact on my antibiotic prescribing decisions |  |  |  |  |  |
| **In my primary care network …** |  |  |  |  |  |
| … antibiotics therapy is discussed |  |  |  |  |  |
| … peer exchange about guideline-oriented antibiotics therapy is offered |  |  |  |  |  |
| ... exchange about antibiotic prescribing routines for non-complicated infections is possible |  |  |  |  |  |
| ... there are conventions about antibiotics for non-complicated infections |  |  |  |  |  |
| … training on guideline-oriented antibiotics therapy is offered |  |  |  |  |  |
| ... I participated in training on guideline-oriented antibiotics therapy |  |  |  |  |  |

| **C. Prescribing medication** |
| --- |

| **When caring for patients with non-complicated infections, I feel obliged to prescribe antibiotics …** | **Strongly disagree** | **Disagree** | **Neutral** | **Agree** | **Strongly agree** |
| --- | --- | --- | --- | --- | --- |
| … because of the patients |  |  |  |  |  |
| ... because of my peer physicians |  |  |  |  |  |
| … because of my former experiences |  |  |  |  |  |
| … because of current literature |  |  |  |  |  |

| **Prescribing antibiotics to patients with non-complicated infections generally leads to** |  |  |  |  |  |
| --- | --- | --- | --- | --- | --- |
| … me having the feeling of being a caring physician |  |  |  |  |  |
| ... me having the feeling of being a competent physician |  |  |  |  |  |
| … a reduced duration of the consultation |  |  |  |  |  |
| ... a reduced frequency of consultations |  |  |  |  |  |
| ... a faster ease of symptoms |  |  |  |  |  |
| … a faster return to work for these patients |  |  |  |  |  |
| … those patients asking for a treatment with antibiotics next time hey have an infection |  |  |  |  |  |
| … these patients feeling more satisfied with my treatment |  |  |  |  |  |
| … these patients seeing a different physician who does not prescribe antibiotics |  |  |  |  |  |
| ... these patients seeing a natural health professional who does not prescribe antibiotics |  |  |  |  |  |

| **D. Allgemeine Angaben zur Person und Praxis** |
| --- |

| Your year of birth? |  (Please enter a year here) | | |
| --- | --- | --- | --- |
| Sex? | female | | male |
| Your medical specialty? |  | | |
| How many professional years of experience as a physician do you have? | ca. years | | |
| In which year did you establish your practice? |  (Please enter a year)  n.a. | | |
| Your employment status? | full-time  part-time | self-employed  employed | |
| What is the size of the community where your practice is located? | population < 5.000  population 5.000 to under 20.000  20.000 to under 100.000  population 100.000 to under 500.000  population > 500.ooo | | |
| In which type of practice are you working? | single practice  group practice | | shared rooms  medical center |
| How many other physicians work in your practice? |  none colleagues  of those full-time part-time  of those physicians in specialty training | | |
| How many medical assistants are working in your practice? |  full-time part-time | | |
| How many patients do you see per quarter on average? | < 500  500-1000 | | 1001-1500  > 1500 |

| Estimated percentage of patients with migrant background in your practice? |  % of patients |
| --- | --- |
| Estimated percentage of patients in your practice, who receive welfare benefits regularly? |  % of patients |

| When did you join the primary care network? |  (Please enter a year) | |
| --- | --- | --- |
| How often do you participate in network events? |  per year (Please enter a number) | |
| How often do you have a communicative exchange with your peer physicians outside network events? | not at all  once a year | once per quarter  several times per quarter |
| Have you implemented any changes in your practice within the last two years (processes, organisation, Organisation, routines)? | yes | no |

**Thank you very much for your participation!**
